# Giant Isolated Omphalocele: Role of Prenatal Diagnosis in Prognostic Asessment and Perinatal Management

**DOI:** 10.1155/2020/4578912

**Published:** 2020-06-09

**Authors:** A. M. Cubo, M. V. Lapresa Alcalde, I. Gastaca, M. O. Rodríguez-Martín, M. C. Martín Seisdedos, M. V. R. Velasco Ayuso, R. C. Cebrián Muiños, J. M. Sayagués

**Affiliations:** ^1^Department of Obstetrics and Gynecology, Complejo Asistencial Universitario de Salamanca and IBSAL, Salamanca, Spain; ^2^Department of Genetics, Complejo Asistencial Universitario de Salamanca, Salamanca, Spain; ^3^Department of Pediatric Surgery, Complejo Asistencial Universitario de Salamanca, Salamanca, Spain; ^4^Department of Hematology, Complejo Asistencial Universitario de Salamanca and IBSAL, Salamanca, Spain

## Abstract

Omphalocele is a congenital malformation of the abdominal wall consisting of a protrusion of the abdominal contents at the base of the umbilical cord. It has a high association with genetic and structural defects; however, if the latter is ruled out, its prognosis improves significantly. Prenatal diagnosis has a key role in this condition as omphalocele can be diagnosed by ultrasound in the first trimester scan, enabling a coordinated approach strategy to achieve the best perinatal results. We present a case report of a pregnant patient with a fetus having a giant omphalocele in which prenatal diagnosis played a decisive role, allowing the coordination of a multidisciplinary team, which was crucial in the immediate care of the newborn.

## 1. Introduction

Omphalocele is a congenital malformation of the abdominal wall in which abdominal contents protrude into a thin-walled sac outside of the abdominal cavity. The protrusion occurs at the base of the umbilical cord and can contain the intestine and/or liver. The estimated prenatal prevalence ranges from 1/3000–1/5000 gestations although postnatal prevalence drops to 0.8/10000 live newborns due to its high association with both genetic and other structural defects, leading to a high rate of spontaneous intrauterine deaths or terminations [1–3]. Its presence is physiological until the 10th week of gestation. The persistence of an omphalocele beyond 12th week is associated with an increase of chromosomal abnormalities, including trisomies 18 and 13 and Beckwith–Wiedemann syndrome. When only the intestine is included, the prevalence of chromosomal abnormalities is four times higher than when the liver is included [3, 4]. On the contrary, when only the liver is included, omphalocele has been associated with worse prognosis due to its greater association with malformations and the increased risk of pulmonary hypoplasia after birth [5, 6]. The presence of isolated omphalocele without association to genetic defect or associated structural anomaly is estimated in 3–6.5% of all cases when it is prenatally diagnosed [3, 7]. Prenatal diagnosis has a key role, as these newborns are a selected population that can benefit from adequate prenatal care and individualized surgical treatment [7], improving their survival.

The size of the omphalocele is also important. Although there is no consensus on the prenatal classification, a giant omphalocele is generally considered when the sac is larger than 5 cm [8, 9]. The relevance of this assessment lies in the further associated complications: the larger the hernia, the greater the likelihood of pulmonary hypoplasia, abdominal compartment syndrome, and surgical repair challenge. Whilst this is mainly a clinical diagnosis, prenatal ultrasound has a role as the estimation of the intrauterine size of the defect and the relationship between its size and the fetal abdomen can help to predict the type of surgical closure [10].

Omphalocele surgical treatment can be primary, staged, or delayed repair, depending on its size and content, the size of the baby, and the intra-abdominal pressure, which must be monitored during surgery to avoid abdominal compartment syndrome [8]. Primary treatment is that performed in the first hours or days of life and can be done using the patient's own tissues (muscles, skin, etc.). Staged repair is used when the defect is too large to be closed by a primary closure; usually this surgery is performed in more than one step and Silo bags, tissue expanders, or meshes are used to enhance the volume of the abdominal cavity to host omphalocele content. Finally, delayed repair is that carried out months or even years after birth; it is based on the transformation of the omphalocele membrane into a neo-skin containing the sac [8, 11]. When possible, early surgical closure is the most appropriate treatment [8, 12]. A coordinated multidisciplinary team including Prenatal Diagnosis, Obstetrics, Neonatology, Pediatric Surgery, and Anesthesiology is essential to achieve success in the management of these patients [13].

## 2. Case Report

**Figure 1:**
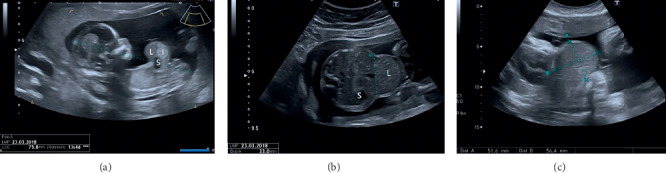
Ultrasound images of the omphalocele. (a) 13 + 4 weeks ultrasound: in the first trimester, the omphalocele contained the liver (L), the intestine (I), and part of the stomach (S). (b) 33 weeks ultrasound: the content of the omphalocele became only hepatic (L), being the stomach (S) correctly situated at the left side of the abdomen. (c) 37 weeks ultrasound: estimation of the final size of the omphalocele before the elective caesarean section.

**Figure 2:**
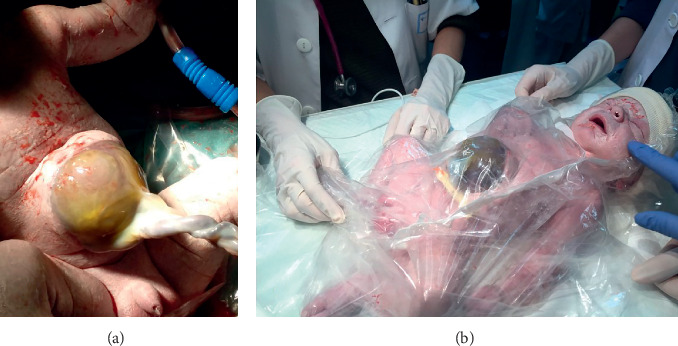
Elective cesarean section. (a) Appearance of the omphalocele at birth. (b) Immediate neonatal care: placement of a thermal bag covering the whole baby's body.

**Figure 3:**
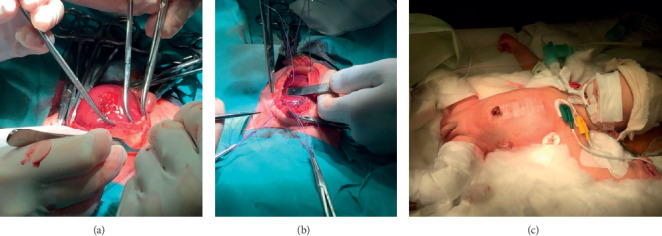
Surgical intervention. The total hepatic content of the omphalocele (a) is visualized. Surgical repair by primary closure of the defect (b). Image of the newborn after intervention (c).

**Figure 4:**
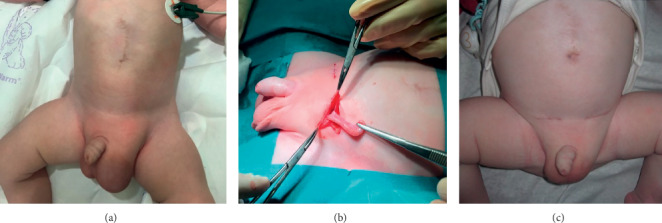
Revision at 3 months of age showing new bilateral inguinal hernia (a). Left hernia repair: the ileocecal junction and the appendix were observed inside (b) and appendicectomy was performed. Image of the baby's abdomen at 4 months of age (c).

We present a clinical case report of a 34-years-old pregnant woman with a single gestation. An omphalocele enclosing mixed intestinal and hepatic content was diagnosed at 12th week (Figure 1(a)). Nuchal translucency (NT) was normal, and no other associated malformations were observed. Parents were counseled regarding ultrasound findings. A genetic study was performed including karyotype, 60K Array-CGH, and methylation-sensitive multiplex ligation probe analysis to analyze the presence of epigenetic and genetic changes related to BeckwithWiedemann Syndrome (BWS), all of them being negative. Parents were informed of the results, as well as the impossibility of ruling out BWS completely despite the negativity of the genetic study, since this disorder is caused by epigenetic defects and there are up to 20% of cases whose diagnosis is clinical and therefore postnatal. After counseling, the patient decided to continue with gestation. The 20th week anomaly scan showed no associated malformations, except a slight upward and leftward heart displacement due to diaphragm elevation. Cardiac function and ductal venosus flow were normal throughout gestation and the heart was structurally normal. At 21st week, the omphalocele content became only hepatic (Figure 1(b)). Ultrasound controls were scheduled every 4 weeks to rule out the early onset of fetal growth restriction, as well as other disorders. At 37th week the estimated bag size was 51 × 56 mm (Figure 1(c)). At 38.3 weeks, the patient was admitted because of mild but regular contractions. At 38.4 weeks, a caesarean section was performed with the coordination of the Obstetrics, Neonatology, Anesthesiology, and Pediatric Surgery teams. The newborn weighed 3540 g, Apgar Test 9-10, and pH 7.32 (Figure 2(a)). A thermal bag covering the whole baby's body was placed immediately after the birth to maintain body heat and reduce the risk of infection (Figure 2(b)). The baby was admitted in the Neonatal Ward for management and surgical preparation in the first hours, starting parenteral nutrition after childbirth. At 48 hours of age, he underwent surgery to repair the omphalocele. A primary closure of the herniation was the first choice, though mesh repairing using a synthetic patch was considered depending on the intra-abdominal pressure (IAP), which was monitored throughout the intervention. As IAP was 15 cm H_2_O, a primary closure of the defect was performed (Figures 3(a), 3(b), and 3(c)). The newborn evolved favorably. He remained relaxed with a rocuronium perfusion for 72 hours after surgery. Analgesia with fentanyl and midazolam perfusion was administered for 6 days after surgery. Intubation was removed 5 days after surgery. Oral feeding was introduced from the 6th day of the procedure. Parenteral nutrition could be removed after 15 days of life. Intestinal transit was normal on the 6th day of surgery. At 3 months of age, two inguinal hernias were diagnosed and repaired surgically (Figure 4(a)). During the exploration of the left herniated sac, the ileocecal junction and the appendix were observed inside. Due to this abnormal location, a prophylactic appendectomy was performed (Figure 4(b)). Currently, the baby is one year old and he is alive and well (Figure 4(c)).

## 3. Discussion

Omphalocele is one of the conditions that can always be detected in the first trimester of gestation [4]. This is an important fact, as complementary tests such as genetic studies, echocardiography, and early anomaly scan can be performed, improving accuracy of the prognosis to provide a correct counseling to parents. Within this issue, there are factors linked to worse prognosis, such as the presence of genetic anomalies (including trisomies 18 and 13 and BeckwithWiedemann syndrome) and associated structural malformations, which may be present up to 50% [2, 4, 7]. Conversely, other markers such as the presence of normal NT in the first trimester of gestation are associated with good prognosis [3]. In our case, the NT was normal (1.8 mm) and no other associated malformations were found in the detailed anomaly scan. The hepatic content of the omphalocele has also been associated with poor prognosis; although this kind of omphalocele is less associated to genetic alterations, an increase in morbidity and mortality is reported due to the higher rate of abnormalities associated (especially cardiac), alterations in the amniotic fluid, and an increased risk of pulmonary hypoplasia after birth [5, 6]. Some authors propose to postpone invasive genetic testing until 2nd trimester in the case of normal NT and absence of associated malformations [3]; nevertheless, amniocentesis was performed at 16 weeks with the patient's consent. Karyotype and 60K Array-CGH were normal as well as the methylation-sensitive multiplex ligation probe analysis in order to detect epigenetic and genetic changes related to BeckwithWiedemann syndrome. Considering this result and the absence of associated visible malformations, parents decided to continue the pregnancy, although they were informed that associated malformations may exist up to 30% despite not seeing them in prenatal anomaly scans and that BWS cannot be completely ruled out prenatally, as up to 20% cannot be detected by prenatal tests and require a clinical and thus postnatal diagnosis [2, 14, 15]. There is no consensus on the timing of prenatal ultrasound appointments, and the related literature is scarce. It seems reasonable to schedule appointments every 3-4 weeks to rule out the early onset of fetal growth restriction, as well as other disorders (cardiac, changes in amniotic fluid, etc.) as growth restriction is described to be more common in these gestations, and if it happens, it is also predictive of an increased risk of adverse neonatal outcome [16, 17]. To assess estimated fetal weight in these fetuses can be difficult by the hernia as measurement of abdominal circumference may be inaccurate. Though some different algorithms have been proposed, we used the Hadlock formula placing the calipers around the abdominal wall and avoiding the hernia so that the ultrasound measuring tool drew the estimated circumference without the omphalocele [18]. All schedules were performed by the same two sonographers to minimize measurement mismatch. All ultrasound controls were normal, and no fetal growth restriction was detected. During the following weeks, appointments with the different specialists were arranged in order to coordinate the birth in the best circumstances. Regarding the mode of delivery, there is no consensus either [2, 19]. Though there is no strong evidence that cesarean delivery improves outcome, it is recommended for fetuses with prenatally assessed giant omphaloceles (defined as those with a sac greater than 5 cm) or when omphalocele contains >75 percent of the liver, in an attempt to avoid dystocia, rupture, infection, and hemorrhage [20]. On this basis, a caesarean section was decided due to the ultrasound omphalocele estimated size (5 cm) and liver content and performed at 38.4 weeks with the coordination of Pediatric, Obstetrics, Anaesthetic, and Surgical teams.

Regarding surgery, early surgical closure using patient's own tissues is proposed as the most appropriate nowadays [8, 12, 13], as it has lower infection rate and better chances of early enteral feeding than staged and delayed surgical repair, though there is a higher risk of abdominal compartment syndrome due to the increase of abdominal pressure after closure. In order to predict and plan the best surgical procedure, different prenatal indices have been proposed, such as the relationship between fetal biometric measurements like abdominal circumference and the diameter of the omphalocele [10, 21]. However, other authors argue that only the attempt to perform a primary closure will allow to assess whether this is possible or not [8, 9]. The feasibility of a primary closure is established postnatally by the measurement of intra-abdominal pressure, which must be monitored during the intervention to avoid abdominal compartment syndrome. An intra-abdominal pressure of less than 20 mm of water will allow a direct closure [8, 22]. Intra-abdominal pressure should be also monitored the days following the intervention in order to early diagnose the onset of postoperative abdominal compartment syndrome [8, 22]. In our case, intestinal transit was restored 6 days after surgery and postoperative intra-abdominal pressure was normal. The baby was discharged 21 days after surgery, with oral nutrition and normal bowel rhythm. The two new inguinal hernias appearing at three months of age are attributable to increased intra-abdominal pressure after the defect closure; their correction was successful and there were no associated complications.

Most of the evidence supports that prenatal diagnosis allows the detection of selected patients with better prognosis enabling intervention planning, which includes the organization of multidisciplinary teams to improve the survival and quality of life of these children [3, 7, 23, 24]. However, some authors state that not only it does not change the prognosis but also increases prematurity, decreases birth weight by ending pregnancy at earlier ages, and increases the rate of caesarean sections and interventionism [2, 25]. We agree with the first statement: despite the hepatic content of the omphalocele, the case we are presenting represents one of the best a priori prognosis events (isolated omphalocele with negative genetic study and absence of associated malformations). The fact of being able to prenatally know these characteristics enabled us to do accurate parents counseling, coordinate a dedicated multidisciplinary team, and plan the end of gestation and the immediate surgical intervention in the best way for the newborn. It was also helpful for the parents who had to take important and difficult decisions; we should keep in mind that despite the good prognosis expected in some cases, omphalocele has a high rate of terminations. For this reason, we believe that prenatal diagnosis improves survival and reduces the morbidity in newborns with this condition.

## 4. Conclusions

- Prenatal diagnosis has a major role in counseling, follow-up, and treatment of omphalocele, since if chromosomal alterations and associated malformations are ruled out, adequate and early postnatal surgical management is possible which significantly improves neonatal prognosis.
- Early surgical closure is the treatment of choice at present. Intra-abdominal pressure is the crucial factor in deciding the type of closure to avoid abdominal compartment syndrome.
- A coordinated multidisciplinary team is a key factor to achieve a successful management of these patients.
